# The identity of crop pollinators helps target conservation for improved ecosystem services^☆^

**DOI:** 10.1016/j.biocon.2013.11.001

**Published:** 2014-01

**Authors:** M.P.D. Garratt, D.J. Coston, C.L. Truslove, M.G. Lappage, C. Polce, R. Dean, J.C. Biesmeijer, S.G. Potts

**Affiliations:** a Centre for Agri-Environmental Research, School of Agriculture, Policy and Development, University of Reading, UK; b Faculty of Biological Sciences, University of Leeds, Leeds, UK; c Naturalis Biodiversity Center, Leiden, The Netherlands

**Keywords:** Crop pollination, Field beans, Oilseed rape, Ecosystem service, Crop pollinators, Pollinator conservation, Bumblebees

## Abstract

Insect pollinated mass flowering crops are becoming more widespread and there is a need to understand which insects are primarily responsible for the pollination of these crops so conservation measures can be appropriately targeted in the face of pollinator declines. This study used field surveys in conjunction with cage manipulations to identify the relative contributions of different pollinator taxa to the pollination of two widespread flowering crops, field beans and oilseed rape. Flower visiting pollinator communities observed in the field were distinct for each crop; while field beans were visited primarily by a few bumblebee species, multiple pollinator taxa visited oilseed, and the composition of this pollinator community was highly variable spatially and temporally. Neither pollinator community, however, appears to be meeting the demands of crops in our study regions. Cage manipulations showed that multiple taxa can effectively pollinate both oilseed and field beans, but bumblebees are particularly effective bean pollinators. Combining field observations and cage manipulations demonstrated that the pollination demands of these two mass flowering crops are highly contrasting, one would benefit from management to increase the abundance of some key taxa, whilst for the other, boosting overall pollinator abundance and diversity would be more appropriate. Our findings highlight the need for crop specific mitigation strategies that are targeted at conserving specific pollinator taxa (or group of taxa) that are both active and capable of crop pollination in order to reduce pollination deficits and meet the demands of future crop production.

## Highlights

• Field beans are pollinated by a few important bumblebee species.
• Oilseed rape is pollinated by a diverse insect community which varies spatially and temporally.
• Managment to improve pollination service should be targeted at specific crops.

## 1 Introduction

Insect pollinators are important for the production of many fruit, vegetable and field crops (Klein et al., 2007) and their contribution to global agriculture has been valued at €153bn annually (Gallai et al., 2009). Like many European countries, insect pollination underpins some key sectors of UK agriculture, particularly the top and soft fruit industries but with increasing areas of insect pollinated field crops such as field beans and oilseed rape being grown, the current valuation of UK insect pollination services of £430 million per annum is set to increase (Smith et al., 2011). Driven by increasing demand for biofuels, the area of oilseed rape in the UK has increased by over 150,000 ha in the past decade (DEFRA, 2012) and with global coverage currently standing at 33.6 million ha (FAOStat, 2013), oilseed is rapidly becoming a crop of global significance.

Pollination of oilseed rape (*Brassica napus*) occurs through a combination of wind and insect vectors with considerable autogamy apparent (Delaplane and Mayer, 2000). Field and cage studies have shown positive effects of insect pollination on pod set and seed set (Jauker and Wolters, 2008; Manning and Wallis, 2005; Morandin and Winston, 2005; Stanley et al., 2013; Williams et al., 1987), with associated benefits to the yield and quality of production (Bommarco et al., 2012). These benefits are dependent on variety and the genetic origin of the oilseed, with some varieties showing increased yield responses to insects (Hudewenz et al., 2013; Steffan-Dewenter, 2003). Overall the contribution of insect pollination to oilseed production has been estimated to be around 18% of total yield (Bommarco et al., 2012).

Oilseed rape is visited by a variety of pollinating insects worldwide, including honey bees, solitary bees and hoverflies (Ali et al., 2011; Arthur et al., 2010). In the UK, bumblebees and honey bees were found to be active flower visitors in oilseed fields (Hayter and Cresswell, 2006) as well as *Andrena* spp., *Osmia* spp*.* and *Lasioglossum* spp*.* solitary bee species (Woodcock et al., 2013). The pollination efficiency of different taxa for oilseed has been shown to vary, *Osmia bicornis* increased pod set when compared to hoverflies (Jauker et al., 2012) and considerable variation between bee species in Pakistan was found (Ali et al., 2011). Furthermore, pollinator behaviour on oilseed flowers in terms of stigma contact and time spent foraging varies (Woodcock et al., 2013), and the amount of pollen carried by different oilseed flower visiting insects depends on taxa (Stanley et al., 2013). Given the variety of wild insects that visit oilseed flowers, and their potential impact on crop production, our understanding of the actual contribution of different taxa to crop pollination in the wider landscape of the UK remains limited.

With a total production area of 168,000 ha in the UK in 2010 (DEFRA, 2012) and 2.3 million ha grown worldwide (FAOStat, 2013), another important insect pollinated field crop is the field bean (*Vicia faba*). The positive effects of insect visits on the pollination of field beans has long been appreciated (Free, 1993), with associated increases in pod set, beans per pod and pod weight; positive impacts on pod distribution on the plant have also been observed (Aouar-Sadli et al., 2008; Benachour et al., 2007; Kendall and Smith, 1975). It has been suggested that only long-tongued bumblebees can access nectar due to the floral anatomy (Free, 1993) but legitimate visitation by honey bees (Kendall and Smith, 1975) and solitary bee species have been observed, although raiding behaviour by some bumblebee species and honey bees is common (Aouar-Sadli et al., 2008; Benachour et al., 2007; Tasei, 1976). Pollinator communities visiting bean flowers in the field have been characterised more recently in North Africa (Aouar-Sadli et al., 2008; Benachour et al., 2007), and in 1976 in France, honey bees, bumblebees and several solitary bee species were observed visiting field beans with varying proportions of legitimate and raiding visits (Tasei, 1976). A systematic survey of field bean visitors and their relative contribution to pollination in the UK has not been undertaken.

In the UK, there is increasing demand for insect pollination services, particularly as field crops reliant on wild pollinators, like oilseed rape, become more widespread (Breeze et al., 2011). With the continued decline of potential insect crop pollinators, both wild (Biesmeijer et al., 2006; Carvalheiro et al., 2013; Potts et al., 2010a) and managed (Potts et al., 2010b), possible associated impacts on production are a concern. If pollination services are to be sustainably managed to maintain crop productivity in the face of increasing demand and continued pollinator decline, it is essential that we identify those pollinators key to production of our most widely grown insect pollinated crops and quantify whether their activity in the field is adequate. Only then can pollination service management strategies be targeted at appropriate species in order to stabilise and improve crop production.

The aims of the present study were to use field surveys to identify insect pollinators which are floral visitors of two important UK flowering crops, field bean and oilseed, as well as establishing their relative level of activity in the field. Then, by using cage manipulation experiments, measure the crop pollination effectiveness of potentially important insect pollinators, thereby identifying those taxa that are currently primarily responsible for crop production and whether their activity in the field is meeting the demands of the crop. This is essential information to underpin pollination service management strategies for safeguarding crop production in the future.

## 2 Materials and methods

### 2.1 Pollinator communities of field bean and oilseed rape

For each crop, pollinator surveys were carried out in eight fields at least 2 km apart. Acknowledging that landscape structure affects the composition of pollinator communities (Kennedy et al., 2013), we used Corine Land Cover map (European Environment Agency, 2010) to characterise the local landscape and chose sites along a gradient of semi-natural habitat. Field bean fields varied between 0% and 46% semi-natural at a 2 km radius and oilseed varied between 0-37%. This ensured that the pollinator surveys in each of our crops would provide a good reflection of the variation in pollinator communities that might be expected in the wider landscape. In each field, two 150 m crop tramlines were selected at least 50 m from the field edge. At 50 m intervals along each tramline, three crop watch areas were established measuring 2 m by 1 m in bean fields and 2 m by 2 m in oilseed rape. At each location, 15 min crop watches were carried out three times during the season, at early, mid and late flowering. All floral insect visits, as well as the number of open flowers, within the crop watch area were recorded. Flower visitors were divided into five taxa: honey bees, bumblebees, solitary bees, hoverflies and others (which included other Diptera, Lepidoptera, Hymenoptera and Coleoptera). Where possible, pollinators were identified to species, and in beans, whether the visitor was nectar raiding or carrying out legitimate visits was recorded. Surveys were carried out only when temperatures were in excess of 15 °C and with no more than light wind. Flowering occurred throughout May for field beans and from the end of April to the end of May for oilseed. Field bean surveys were undertaken in 2011 in Berkshire on winter sown field beans, variety Wizard. Oilseed surveys were carried out in 2012 in Yorkshire on the restored hybrid varieties Excalibur and DK Expower.

### 2.2 Effect of different pollinators on field bean and oilseed rape pollination

To enable manipulation of both flowering crops and pollinators, flight cages were constructed at the University of Reading and University of Leeds experimental farms, using 2.4 by 2.1 m frames covered in polyethylene mesh with a gauge size of 1.33 mm. In separate flight cages, four potential crop pollinators were established: honey bees (*Apis mellifera*), bumblebees (*Bombus terrestris-audax* – a UK subspecies)*,* a solitary mason bee (*O. bicornis*) and a hoverfly (*Episyrphus balteatus*). These pollinators were chosen because they are commercially available and represent four distinct flower visiting insect guilds which may be effective crop pollinators. Pollinators were provided with nesting and forage resources within the cage when not involved in experiments, thus encouraging natural foraging behaviour for the period of experimentation. *Apis mellifera*, through the use of a double entrance hive, was also given access both to the flight cage and the outside.

To compare the effects on pollination of our four pollinator species, flowering oilseed rape and bean plants were placed in a randomised block in flight cages with pollinators for a controlled number of visits per flower. Oilseed rape (cultivar: Heros) and field bean (cultivar: Clipper) plants were grown individually in pots. Experimental plants were planted in multiple temporal cohorts to ensure plants at the correct phenological stage were available for pollinator treatments and to enable repeated experimentation through time. During pollinator exposures, cages contained either 3 bean plants, or 10 oilseed plants, of which 5 were experimental. Within the cage, a focal plant was selected at random and all flower visits to that plant were recorded until a threshold number of visits was reached. By incorporating the total number of flowers within the cage, pollinator visitation rates to experimental plants could be manipulated by controlling the length of time plants were inside cages. Experimental visitation rates used were 1 (low) and 3 (high) visits on average per flower for oilseed, and 1 (low), 2 (medium) and 4 (high) visits on average per flower for field bean. Following exposure to pollinators, all flowers in anthesis on each of the experimental plants were marked with cable ties. Due to potential effects of plant phenology on responses to pollination, only oilseed plants which had any of the first 30 flowers on the main stem open and field beans, in flower up to node 11, were used for experiments.

The availability of plant cohorts at the appropriate phenological stage, in conjunction with active pollinators within flight cages meant that two bean cohorts per year were involved in the study and from these, nine bumblebee, seven honey bee, five mason bee and six hoverfly replicates of high, medium and low visitation rates were possible. Eight oilseed cohorts were utilised, from which nine bumblebee, eight honey bee, eight mason bee and six hoverfly replicates of high and low visitation rates were carried out.

In addition to insect pollinator treatments, for each crop cohort a series of additional treatments were set up. Ten plants from each cohort were randomly selected and assigned, in groups of 5, either a hand pollination or pollinator excluded treatment. For oilseed hand pollinated plants, the first 30 flowers to develop on the primary stem were supplementary pollinated, with pollen from 5 donor plants. For beans, hand pollination on all flowers on two or three consecutive nodes, between nodes 1 and 11 on one stem of each plant was done using pollen from two donor plants. For the pollinator exclusion treatments, the five randomly selected plants from each cohort were stored in isolation cages for the duration of flowering.

Before and after pollinator exposure, plants were stored, by cohort, in randomised blocks within isolation cages and allowed to mature and ripen naturally. Hand pollinated and pollinator excluded plants were stored with their respective cohorts. At harvest, the number of bean pods per node and for oilseed, the number of set and failed pods from experimentally manipulated flowers (those marked with cable ties), was noted. Field bean pods then received further drying for 48 h in an 80 °C oven. The number of beans per pod was recorded and beans were weighed to the nearest 0.001 g. For oilseed, five randomly selected experimentally manipulated pods from each plant were collected. The number and weight, again to the nearest 0.001 g, of all seeds in those pods was recorded.

### 2.3 Analysis

An average visitation rate (visit per flower per minute) was calculated across the 6 crop watch locations of each field for each survey round. Analysis of variance was used to analyse the influence of pollinator taxa, survey round, site and the pollinator:survey round interaction on visitation rate. Models were then simplified until only pollinator taxa and any other significant effects remained. If there was a significant effect of pollinator taxa on visitation rate then a Tukey honest significant difference test was used to determine significant differences between pollinator taxa. Visitation data was log + 1 transformed to improve normality prior to analysis.

Linear mixed effects models were used to analyse pollinator and visit number effects on bean pods per node, beans per pod, bean weight and pod weight. Pollinator, visit number (L, M, H) and their interaction were included in the model as fixed effects; Year (2011, 2012), University (Reading, Leeds) and replicate within cohort (1–4) were random effects. Models were then simplified to include only significant fixed effects. To improve normality, pods per node was log + 1 transformed prior to analysis. Plants which produced no pods on the treatment nodes were removed from the bean number, bean weight and pod weight analysis. Linear mixed effects models were also used to investigate pollinator and visit number effects on seeds per pod, seed weight and pod weight for oilseed. Pollinator, visit number (L, H) and their interaction were included in the model as fixed effects with University (Reading, Leeds), cohort (1–8) and replicate within cohort (1–3) as random effects. Due to non-normal data, seed weight was log transformed prior to analysis. Pod set represents the proportion of flowers exposed to pollinators that set pods. A generalised linear mixed effects model with a binomial error structure and the same fixed and random effects was used to analyse pod set.

To compare hand pollinated and pollinator excluded treatments with insect pollinator treatments, mixed effects models were used again for each of the yield parameters for both beans and oilseed. In this case, pollination treatment only was included as a fixed effect and the separate visit number replicates were included in the model as an additional random effect nested in replicate. All analysis was carried out using R version 2.14.1.

## 3 Results

### 3.1 Pollinator communities of field bean and oilseed rape

**Fig. 1:**
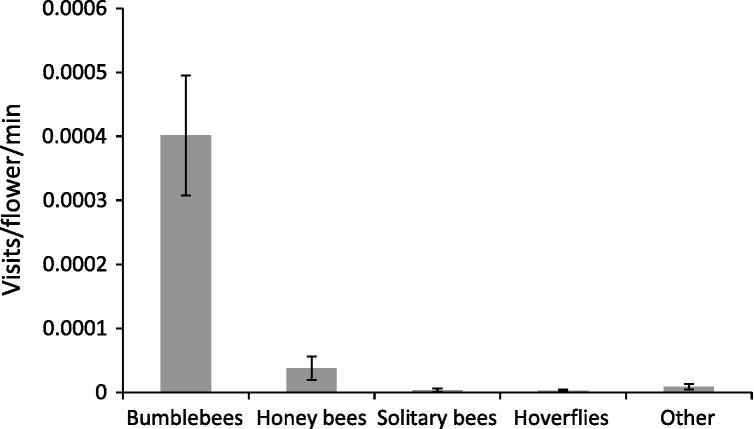
Visits/flower/minute shown by some potentially important pollinator taxa legitimately visiting field beans across eight field sites in Berkshire, UK. Mean ± S.E.M.

All pollinator taxa were observed visiting beans on at least one occasion. Of those bumblebees that were positively identified, 54% of legitimate visits were made by *B. terrestris/lucorum*, 19% by *B*. *hortorum*, 17% by *B*. *lapidarius*, 8% by *B*. *pascuorum*, 1% by *B*. *hypnorum* and less than 1% by *B*. *pratorum*. In addition to these legitimate visits, a number of bee species were recorded raiding floral nectar through the back of the flower. Eighty-three percent of visits by *B*. *pratorum* were raids*,* 50% of *B*. *hypnorum*, 44% of *B*. *terrestris/lucorum*, 29% of *B*. *lapidarius*, 10% of *B*. *pascuorum*, 2% of *B*. *hortorum* and 23% of visits by honey bees were raids. Legitimate visits per flower per minute by bumblebees was significantly higher than all other pollinator taxa (*F*_4-115_ = 16.61, *P* = <0.0001) (Fig. 1). There was no significant effect of survey round (*F*_2-106_ = 0.081, *P* = 0.92), field site (*F*_7-108_ = 2.07, *P* = 0.053) or a pollinator:round interaction (*F*_8-98_ = 0.078, *P* = 1.0) on insect visitors.

**Fig. 2:**
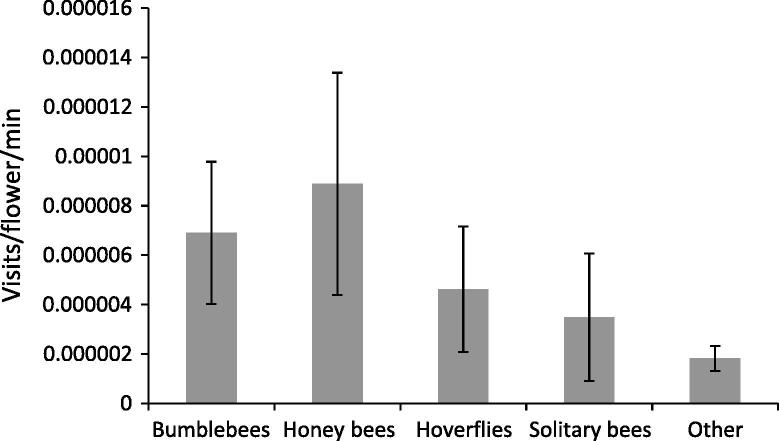
Visits/flower/minute shown by some potentially important pollinator taxa visiting oilseed rape across eight field sites in Yorkshire, UK. Mean ± S.E.M.

All study taxa were observed visiting oilseed flowers. There was no significant difference in the visitation rates of different pollinator taxa in oilseed fields (*F*_4-98_ = 1.17, *P* = 0.33) (Fig. 2). There was a significant effect of site (*F*_7-98_ = 2.96, *P* = 0.0074) and a pollinator:survey round interaction (*F*_8-98_ = 2.50, *P* = 0.016). Overall, there was no significant effect of survey round on visits per flower per minute (*F*_2-98_ = 2.76, *P* = 0.068). A high number of non-syrphid flies were also observed on flowers, although movement between flowers during observations was very rare. The pollination efficiency of these flies is not known and they were not subject to cage manipulations as part of this study, they were therefore excluded from the analysis. Further research is necessary to understand the contribution of other diptera groups on the pollination of oilseed.

### 3.2 Effect of different pollinators on field bean pollination

**Fig. 3:**
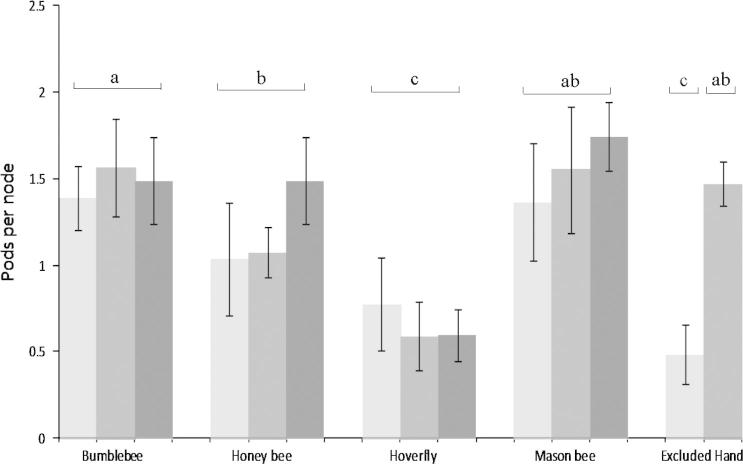
Pods per node on field beans following visitation by four different pollinators at three visitation rates per flower (1 = [], 2 = [], 4 = [] visits). Pods per node following pollinator exclusion and hand pollination also shown, Mean ± S.E.M. Treatments with different letters are significantly different according to a linear mixed effects model.

There was a significant effect of pollinator on pod set (*F*_3-229_ = 11.87, *P* < 0.0001) of beans, with hoverflies setting significantly fewer pods per node than bumblebees, honey bees and mason bees. Pod set by bumblebees was also significantly greater than pod set by honey bees (Fig. 3). There was no significant effect of visit number (*F*_2-227_ = 1.39, *P* = 0.25) or a pollinator:visit number interaction (*F*_6-221_ = 1.21, *P* = 0.30) on pod set. There was no significant effect of pollinator, visit number or a pollinator:visit number interaction on beans per pod (Table 1). Similarly, no significant effect of pollinator, visit number or their interaction on bean weight or pod weight was found (Table 1). There was a significant effect of control treatments (i.e. hand pollination and pollinator exclusion) on pods set per node (*F*_5-244_ = 13.84, *P* < 0.001) with pollinator excluded treatments setting fewer pods than bumblebees, honey bees and mason bees. Hand pollination treatments also resulted in significantly greater pods set than hoverfly pollination (Fig. 3). There was no significant effect of control treatments on beans per pod, bean weight or pod weight (Table 1).

### 3.3 Effect of different pollinators on oilseed rape pollination

**Fig. 4:**
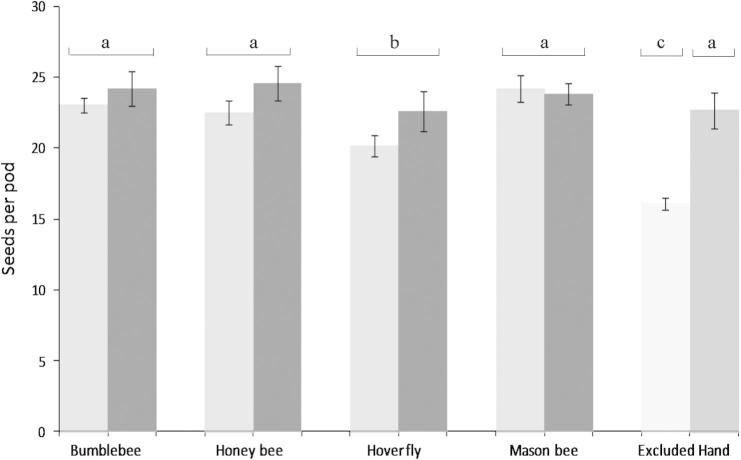
Seeds per pod of oilseed rape following pollination by four different pollinators at two visitation rates per flower ([] = 1, [] = 3 visits). Seed numbers following pollinator exclusion and hand pollination also shown, Mean ± S.E.M. Treatments with different letters are significantly different according to a linear mixed effects model.

There was a significant effect of pollinator (*F*_3-290_ = 7.74, *P* = 0.0008) and visit number (*F*_1-290_ = 7.55, *P* = 0.0064) on oilseed seeds per pod, with hoverflies showing fewer seeds than other pollinators. Lower visitation rates also resulted in fewer seeds per pod. There was no significant pollinator:visit number interaction on seeds per pod (*F*_3-287_ = 1.91, *P* = 0.13) (Fig. 4). There was a significant pollinator:visit number interaction effect on seed weight but no significant direct effects of pollinator or visit number (Table 2). Pod weight was significantly affected by both pollinator and visit number, again with hoverflies and low visitation rates showing the lowest pod weights. Pollination by mason bees also resulted in greater pod weights than pollination by honey bees (Table 2). No significant pollinator:visit number interaction was found. Significantly fewer pods set under low visit numbers but no significant effect of pollinator or a pollinator:visit number interaction on pod set was found (Table 2). There was a significant effect of control treatments on seed number (*F*_5-358_ = 22.72, *P* < 0.0001) with pollinator excluded and hoverfly treatments setting fewer seeds than all other treatments and pollinator exclusion also resulting in fewer seeds than hoverfly pollination (Fig. 4). The same pattern was seen for pod weight, although pod weight following pollination by mason bees was also significantly greater than honey bee and hand pollination treatments (Table 2). No such effect was seen for seed weight. Pod set was affected by treatment, with pollinator excluded treatments significantly lower than all other treatments (Table 2).

## 4 Discussion

### 4.1 Field bean pollination

The vast majority of pollinators carrying out legitimate flower visits in field bean fields in Berkshire were bumblebees. This pattern was common throughout the season and across sites, as indicated by the absence of significant survey round and site effects. The clear prominence of bumblebees visiting beans is perhaps consistent with the morphology of bean flowers limiting access to nectar for smaller solitary bee species and honey bees, and supports conclusions made by Free (1993). Insect visitation improved pod set in beans, and bumblebees, honey bees and mason bees have the capacity to improve pod set by between 60% and 69%. The absence of a significant visit number effect suggests that good pod set is achieved with visitation rates as low as an average of one visit per flower. Bumblebees did increase pod set above that of honey bees indicating that they may be particularly effective field bean pollinators, although such a difference between bumblebees and honey bees were not seen by Kendall and Smith (1975). The inability of the hoverfly, *E*. *balteatus* to pollinate beans is unsurprising given their small size and lack of robustness to carry out legitimate visits. Positive impacts of insect visitation on bean quality in terms of size, reported in earlier studies (Aouar-Sadli et al., 2008; Benachour et al., 2007), was not apparent in this work.

We showed bumblebees are key bean pollinators and this is a product of their high activity in the field and good pollination efficiency. Our field surveys showed an average visitation rate of 0.0004 flowers per minute. As bean flowers remain open to bee visits for 3 days (Osborne et al., 1997) and assuming 8 h of pollinator foraging per day in good weather, this would mean that, on average, 58% of flowers could expect at least one visit. Not all flowers on bean plants will set pods regardless of levels of pollination, and this depends on node location and flower numbers per node (Free, 1993), but 58% of flowers visited is by no means saturation and production could therefore be vulnerable to bumblebee decline or low visitation in poor weather years.

### 4.2 Oilseed rape pollination

Pollinator surveys showed that oilseed rape flowers are visited by a more diverse pollinator community than field beans and there was no significant difference in visitation rates between any pollinator taxa. There was a significant effect of site on visitation and a significant pollinator:survey round interaction. This temporal and spatial variation points to seasonal and local landscape effects on crop visitors. The open and accessible nature of oilseed flowers means they are visited by a diverse pollinator community, one that is more responsive to seasonal and local factors, particularly when compared to the relatively specialised and mobile bumblebees seen in high numbers on beans. This diversity of insect visitors to oilseed has also been seen in other studies and on similar crops, some showing impacts of seasonality and local landscape (Ali et al., 2011; Arthur et al., 2010; Hayter and Cresswell, 2006; Rader et al., 2012; Woodcock et al., 2013). Many non-syrphid flies were observed on oilseed flowers and although their contribution to pollination was not tested in this study, it is important that their potential contribution is quantified in future research, if the pollination ecology of oilseed is to be fully understood.

In common with previous studies, this research highlights the improved pollination of oilseed flowers following insect visitation (Bommarco et al., 2012; Jauker et al., 2012; Stanley et al., 2013), but also highlighted is that very distinct taxa can improve pollination when compared with pollinator excluded treatments. Improved oilseed pollination by bumblebees, honey bees and mason bees when compared to hoverflies was also apparent, with increased seeds per pod. The number of seeds per pod after exposure to these three pollinators was not significantly different from hand pollination treatments, suggesting that these three pollinators are also achieving maximum pollination after as few as three visits on average per flower.

Using field visitation rates for our potential pollinators and assuming oilseed flowers are receptive for 3 days (Bell and Cresswell, 1998), our data demonstrates that in 2012, only 3.4% of oilseed flowers could expect a visit from a pollinator. Given the positive effects of insect visitation on pollination of oilseed, this indicates that insect pollination service in our study fields could be severely limited, particularly when 3 visits is better than 1 with regards to maximising pollination. This has potential negative implications for the yield and quality of UK oilseed (Bommarco et al., 2012) and needs to be addressed through appropriate management of insect pollinator communities.

### 4.3 Conserving pollinators for improved ecosystem services

Driven by habitat loss and falling floral abundance and diversity, Europe and the US have seen significant declines in many bumblebee species (Goulson et al., 2008). Given that six species of bumblebee were recorded visiting beans in the present study, four in significant numbers, declines in any of these species has implications for field bean pollination. The ability of distinct pollinator taxa to improve oilseed pollination, and spatial and temporal variation in field activity of these taxa, demonstrates that the pollination ecology of oilseed is contrasting to that of field beans. Field beans are reliant on a few key pollinators whilst oilseed is serviced by a more diverse and variable pollinator community. Pollinator management strategies to maintain or improve production in each of these crops must therefore be targeted accordingly.

Management to support field bean pollinators should be aimed at maintaining or increasing bumblebee abundance. Despite the proven ability of honey bees and mason bees to pollinate beans, very low activity in the field would suggest resources would be better targeted at bumblebees. The establishment of additional floral resources within agricultural landscapes can increase the local abundance and diversity of bumblebees (Pywell et al., 2011, 2006). Such measures could be implemented to stabilise bumblebee populations or even boost them, improving crop pollination, particularly if flower choice is targeted specifically at those bumblebee species showing potential as good field bean pollinators, namely the long tongued species (Carvell et al., 2011). Our study shows the long tongued *Bombus hortorum* could be a highly effective bean pollinator due to its high activity in the field and low flower raiding activity. While improving local floral resources can help bumblebee populations in the long-term, maximising field bean pollination may require planting species that do not co-flower with beans, or cutting flower margins during bean flowering so encouraging bumblebees onto the crop. The context of any management option in terms of local landscape and agricultural system however, must be considered to maximise its effects (Scheper et al., 2013). Utilisation of commercially available pollinators, as seen for some tree crops and in protected cultivation, could be adopted. The low unit area value of field beans and high cost of commercially produced bumblebees, however, would most likely preclude this as a viable option, thus local and landscape scale habitat manipulation to conserve bumblebees would be more cost effective.

Considering the influences of season and local landscape on oilseed flower visitors, management to support general pollinator diversity would provide stability in oilseed pollination services in the face of ongoing landscape and environmental change. Furthermore, management to increase general pollinator abundance could address the sub-optimal pollinator activity observed in this study. Management of meadows or buffer strips under certain agri-environment schemes have been shown to increase pollinator diversity and abundance, with associated improvement in pollination service, albeit for non-crop species (Albrecht et al., 2007), and sown flower strips not only support bumblebees but also hoverflies (Haenke et al., 2009). Furthermore, natural and semi-natural habitats can benefit pollinator diversity and the stability of pollination service (Garibaldi et al., 2011), and these habitats should be maintained within agricultural landscapes to ensure a diverse and abundant pollinator community for oilseed, although the extent and location of these habitats should be optimised (Brosi et al., 2008). To maximise cost benefit of any pollinator management strategy, the value of pollinator diversity to crop production, through both synergistic effects and buffering of landscape and environmental change, needs to be understood. This research has begun (Brittain et al., 2013a,b; Greenleaf and Kremen, 2006; Hoehn et al., 2008) and the present study further highlights the potential benefits of diverse pollinator communities for crop pollination and in oilseed in particular.

## 5 Conclusion

The proliferation of field grown insect pollinated crops puts new pressures on wild insect pollination services and it would appear, certainly for oilseed that these demands are not currently being met. The importance of insect pollination for crop production is clear but the specific demands of a crop, considering both pollinator activity in fields and the pollination efficiency of those pollinators, is crop specific, thus pollination service management strategies must be targeted. Some crops, such as oilseed, will benefit from management to increase general abundance and diversity of pollinator populations so pollination services can be provided in different landscapes and in changing environments. By contrast, other crops, including field beans, will benefit from more tailored mitigation strategies to increase the abundance of the more functionally important taxa through targeted management of local landscapes.

## Figures and Tables

**Table 1:** Yield measures of field bean following pollination by four different pollinators at three visitation rates per flower (L = 1, M = 2, H = 4 visits). Yield following pollinator exclusion and hand pollination treatments also shown, Mean ± S.E.M. *F* and *P* values for main effects shown, following mixed effects models including pollinators and visit numbers and models including pollinators and control treatments. Means with different letters are significantly different (*P* < 0.05).

Pollinator	Visit number	Beans per pod	Pod weight (g)	Bean weight (g)
Bumblebee	L	2.67 ± 0.16	2.12 ± 0.24	0.78 ± 0.06
	M	2.73 ± 0.18 a	1.20 ± 0.22 a	0.71 ± 0.04 a
	H	2.44 ± 0.21	1.83 ± 0.29	0.65 ± 0.09

Honeybee	L	2.55 ± 0.17	2.08 ± 0.23	0.79 ± 0.06
	M	2.29 ± 0.15 a	1.99 ± 0.10 a	0.91 ± 0.07 a
	H	2.52 ± 0.16	2.00 ± 0.23	0.78 ± 0.05

Hoverfly	L	2.02 ± 0.42	1.74 ± 0.35	0.73 ± 0.15
	M	2.70 ± 0.25 a	2.32 ± 0.26 a	0.77 ± 0.10 a
	H	2.74 ± 0.27	2.07 ± 0.22	0.79 ± 0.09

Mason bee	L	2.56 ± 0.40	1.74 ± 0.08	0.71 ± 0.09
	M	2.51 ± 0.16 a	1.46 ± 0.17 a	0.55 ± 0.04 a
	H	2.73 ± 0.23	1.81 ± 0.22	0.65 ± 0.04

Pollinators and visit number	Pollinator	*F*_3-198_ = 2.09, *P* = 0.10	*F*_3-198_ = 0.88, *P* = 0.45	*F*_3-196_ = 1.17, *P* = 0.32
	Visit number	*F*_2-196_ = 0.50, *P* = 0.60	*F*_2-196_ = 0.06, *P* = 0.94	*F*_2-199_ = 1.69, *P* = 0.19
	Pollinator:visit number	*F*_6-190_ = 0.79, *P* = 0.58	*F*_6-190_ = 0.68, *P* = 0.67	*F*_6-190_ = 1.10, *P* = 0.37

Bumblebee		2.62 ± 0.15 a	2.00 ± 0.19 a	0.72 ± 0.04 a
Honeybee		2.44 ± 0.12 a	2.00 ± 0.16 a	0.83 ± 0.05 a
Hoverfly		2.58 ± 0.15 a	2.02 ± 0.19 a	0.75 ± 0.09 a
Mason bee		2.54 ± 0.04 a	1.64 ± 0.13 a	0.64 ± 0.05 a
Pollinator excluded		2.27 ± 0.20 a	1.97 ± 0.20 a	0.83 ± 0.07 a
Hand pollination		2.82 ± 0.29 a	1.69 ± 0.30 a	0.62 ± 0.14 a

Pollinators and controls		*F*_5-202_ = 1.20, *P* = 0.31	*F*_5-202_ = 0.49, *P* = 0.78	*F*_5-202_ = 1.54, *P* = 0.18

**Table 2:** Yield measures of oilseed rape following pollination by four different pollinators at two visitation rates per flower (L = 1, H = 3 visits). Yield following pollinator exclusion and hand pollination treatments also shown, Mean ± S.E.M. *F*, *Z* and *P* values for main effects shown, following mixed effects models including pollinators and visit numbers and models including pollinators and control treatments. Means with different letters are significantly different (*P* < 0.05).

Pollinator	Visit number	Seed weight (mg)	Pod weight (g)	Pod set%
Bumblebee	L	0.044 ± 0.001	0.102 ± 0.007	95.91 ± 1.74
	H	0.042 ± 0.002 a	0.102 ± 0.005 ab	97.96 ± 0.68 a

Honeybee	L	0.043 ± 0.002	0.096 ± 0.007	94.89 ± 1.57
	H	0.041 ± 0.001 a	0.101 ± 0.004 b	97.07 ± 1.92 a

Hoverfly	L	0.044 ± 0.003	0.087 ± 0.009	97.04 ± 1.09
	H	0.043 ± 0.003 a	0.097 ± 0.008 c	97.41 ± 1.67 a

Mason bee	L	0.044 ± 0.002	0.107 ± 0.007	97.48 ± 1.35
	H	0.048 ± 0.002 a	0.114 ± 0.009 a	97.88 ± 0.95 a

Pollinators and visit number	Pollinator	*F*_6-287_ = 1.51, *P* = 0.21	*F*_3-290_ = 5.99, *P* < 0.001	*Z* < 1.25, *P* > 0.21
	Visit number	*F*_6-287_ = 0.17, *P* = 0.68	*F*_1-290_ = 4.79, *P* = 0.03	*Z* = 3.19, *P* < 0.01
	Pollinator:visit number	*F*_3-287_ = 3.09, *P* = 0.03	*F*_3-287_ = 0.74, *P* = 0.53	*Z* < 1.01, *P* > 0.31

Bumblebee		0.043 ± 0.001 a	0.101 ± 0.006 abc	96.94 ± 1.11 a
Honeybee		0.042 ± 0.001 a	0.098 ± 0.005 b	96.03 ± 1.38 a
Hoverfly		0.044 ± 0.003 a	0.092 ± 0.008 c	97.27 ± 1.18 a
Mason bee		0.046 ± 0.002 a	0.110 ± 0.008 a	97.68 ± 1.02 a
Pollinators excluded		0.044 ± 0.002 a	0.067 ± 0.007 d	83.33 ± 3.43 b
Hand pollination		0.042 ± 0.002 a	0.094 ± 0.005 b	95.98 ± 1.54 a

Pollinators and controls		*F*_5-358_ = 1.20, *P* = 0.31	*F*_5-358_ = 18.73, *P* < 0.001	*Z* > 5.83, *P* < 0.001
